# WHO/INRUD Core drug use indicators and commonly prescribed medicines: a National Survey from Sri Lanka

**DOI:** 10.1186/s40360-021-00535-5

**Published:** 2021-10-28

**Authors:** P. Galappatthy, P. Ranasinghe, C. K. Liyanage, M. S. Wijayabandara, S. Mythily, R. L. Jayakody

**Affiliations:** Department of Pharmacology, Faculty of Medicine, University of Colombo, No 25, Kynsey Road, Colombo, 08 Sri Lanka

**Keywords:** Prescribing indicators, Rational drug use, International network on rational use of drugs (INRUD), Sri Lanka, Private sector pharmacies

## Abstract

**Background:**

Identification of internationally comparable indicators of medicines use are important for a country to implement strategies and regulations to improve usage of medicines. Sri Lanka established a new National Medicines Regulatory Authority in 2015 and this survey evaluated the medication use indicators in Sri Lanka, according to the International Network on Rational Use of Drugs (INRUD), prior to its implementation.

**Methods:**

This descriptive-cross-sectional study was conducted in 80 pharmacies, representing all 25 districts of the country. Three pharmacy categories were included; privately owned pharmacies, ‘Rajya Osusala’ pharmacies operated by the State Pharmaceuticals Corporation (SPC) of Sri Lanka and SPC Franchisee pharmacy outlets. Selection of pharmacies from respective districts were done proportionate to estimated population. Data were collected to identify WHO/INRUD core drug use indicators and the commonly prescribed medicines.

**Results:**

Total of 2328 prescriptions were included (‘Rajya Osusala 559; SPC Franchise 711; private pharmacies 1058). Altogether 7,255 medicines were prescribed, and the 3 most commonly prescribed medicines were atorvastatin, losartan and metformin. Average number of medicines per encounter was 3.1±1.9 (Median: 3; range 1-12) Highest average number of medicines per encounter was reported in prescriptions received at ‘Rajya Osusala’ pharmacies (3.6±2.2), significantly higher than in other categories of pharmacies (*p*<0.001). Percentage of medicines prescribed by generic name was only 35.5%, highest at the ‘Rajya Osusala’ pharmacies (40.6%), significantly higher than other categories of pharmacies. The overall percentage of medicines prescribed from essential medicine list (EML) was 68.8%, without any significant variation between different categories of pharmacies. The percentage of medicines actually dispensed and accurately labelled were 92.4 and 98.5% respectively.

**Conclusions:**

The average number of medicines per encounter was higher than the WHO recommended value but the usage of antibiotic and injectable drugs were within recommended standards. Generic prescribing, was very much lower. The EML prescribing, labelling and percentage dispensed medicines fared much better although lower than the WHO recommended 100% compliance. This island wide study has provided national wide data before the implementation of key changes in regulation of medicines in Sri Lanka and a repeat survey will be useful to identify impact of the new legislations.

**Supplementary Information:**

The online version contains supplementary material available at 10.1186/s40360-021-00535-5.

## Background

Health care quality is the degree to which health care services provided by an institution improves desired outcomes. This is determined by comparison with set standard measurements and indicators. Assessment of health care quality is gaining broader significance, and it is assessed based on the provision of safe, effective, efficient, timely and equitable health care. Rational use of medicines is an essential element in ensuring the quality of health care for patients and the community [1]. It is defined by the World Health Organization (WHO) as patients receiving medications appropriate for their clinical requirements, in doses that meet their individual needs for an adequate period of time, at the lowest cost to them and their community [2]. Rational use of medicines is closely aligned with effectively curing a disease, relieving the symptoms of disease, or the goal of prevention and prophylaxis through therapy based on scientific evidence and well-studied clinical guidelines [3].

Studies have shown that irrational use of medicines is seen in both developed [4, 5] and developing countries, albeit more common in developing countries [6–8] around the world. The common types including polypharmacy, inappropriate use of anti-microbials and prescribing medicines without adhering to clinical guidelines [9]. Irrational use is associated with an increased incidence of adverse effects, medication interactions and the emergence of antimicrobial resistance [10]. Furthermore, it also contributes to a substantial increase in cost to health care around the world [11]. Identifying the magnitude of irrational use is an essential step towards instituting corrective measures to promote rational prescribing. To achieve this objective the WHO in collaboration with the International Network of Rational Use of Drugs (INRUD) has developed core drug use indicators to evaluate and compare practices in health care settings, which includes indicators on prescribing, patients-care and health care facilities [12].

Sri Lanka, is an island nation in the South Asian region that has a population of nearly 22 million [13]. Although a low-middle income country, Sri Lanka is often recognized internationally for its commendable health indicators at a comparatively low level of Gross Domestic Product (GDP) and it is arguably in the forefront in the provision of quality health services in the region [14]. Sri Lanka has a universal health care system that extends free healthcare to all citizens via a network of over 500 public healthcare institutions/hospitals scattered throughout the country. Fee-levying private hospitals and general practitioners (GPs) also provide a share of all inpatient/outpatient services [15]. It is estimated that the private sector accounts for between 50 and 60% of out-patient care [16]. Prescription medicines issued in the public sector are dispensed free-of-charge via pharmacies available at those institutions/hospitals. However, patients will need to rely on a fee-levying pharmacy of their choice for prescriptions issued in the private sector and/or when a particular prescribed medication is not available in the relevant public sector pharmacy. Expenses for such prescriptions need to be entirely born by the patient, with insurance payments available to the few who have subscribed to a self-paid voluntary health insurance scheme or for those who are offered a health insurance policy as part of their employment. These fee-levying pharmacy outlets belong to one of three categories based on ownership, a) privately owned pharmacies, b) ‘Rajya Osusala’ (state owned pharmacy outlets) operated by the State Pharmaceuticals Corporation (SPC) of Sri Lanka and c) SPC Franchisee pharmacy outlets. Previous studies have not observed marked differences in pricing between the state owned ‘Rajya Osusala’ pharmacies and privately owned pharmacies [16].

Irrational use of medicines is quite prevalent in the country with small scale regional studies indicating the presence of a high degree of polypharmacy, irrational use of anti-microbials and self-medication [17, 18]. To date there are no nationally representative surveys exploring the problem of irrational medicine use in Sri Lanka. In 2015, the Sri Lankan government established a new National Medicines Regulatory Authority (NMRA) and a new NMRA Act for regulation of medicines. The purpose of this study was to describe the WHO/INRUD core drug use indicators in a nationally representative sample of private sector pharmacies in all twenty-five administrative districts of Sri Lanka and describe the pattern of medication use indicators before the implementation of the NMRA Act of 2015. The findings of this study would help policy makers understand rational medication use practices in a developing country and help to identify the impact of new regulations. It would also provide useful information to device and implement further appropriate actions to promote rational use of medicines.

## Methods

### Study setting

This descriptive cross-sectional study was conducted in 80 pharmacy outlets, representing all 25 districts in the country between July to September 2015. The 80 pharmacy outlets belonged to all three categories of pharmacies based on type of ownership currently operational in Sri Lanka. These were a) privately owned pharmacies, b) ‘Rajya Osusala’ (state owned pharmacy outlets) operated by the State Pharmaceuticals Corporation (SPC) of Sri Lanka and c) SPC Franchisee pharmacy outlets. Selection of pharmacy outlets from each of the 25 districts were done proportionate to the estimated population, following the WHO/Health Action International (HAI) methodology [19]. According to the WHO/HAI methodology each survey area (district) should at least cover a population of 100,000. However, the districts of Mannar and Mullaitivu did not have the minimum required population and were considered together as one survey area. In Districts where the population exceeded 1 million (Colombo, Gampaha, Kandy, Kurunegala, Kalutara, Rathnapura and Galle), 2 sets of samples were collected, depending on the availability of the different categories of pharmacy outlets mentioned above. The district wise number of pharmacy outlets included in the study are depicted in Supplementary Table S1. It is important to note that ‘Rajya Osusala’ pharmacies and/or SPC Franchisee pharmacy outlets were not operational in all of the districts (Supplementary Table S1).

The latest lists of ‘Rajya Osusala’ outlets and SPC franchise pharmacies was obtained from the SPC and the list of registered private pharmacies were obtained from the National Medicines Regulatory Authority (NMRA) of Sri Lanka. According to the grading of public hospitals in Sri Lanka, the main public hospital in each survey district was selected as a landmark to select pharmacies for the study. Pharmacy outlets, one from each category (depending on availability) situated within 3 km of landmark hospital was selected randomly for the study. Ethics approval for the study was obtained from the Ethics Review Committee, Faculty of Medicine, University of Colombo (ERC-15-189) and institutional approval was obtained from Faculty of Medicine, University of Colombo for data collection and analysis. Permission was also obtained from the Ministry of Health, National Medicines Regulatory Authority (NMRA), the SPC and proprietors of the private sector pharmacy outlets. All methods were performed in accordance with the relevant guidelines and regulations.

### Study design, definitions and outcome measures

Data were collected to identify WHO/INRUD core drug use indicators and commonly prescribed medicines in the above study settings. According to WHO recommendations, at least 600 encounters should be included in a cross-sectional survey in order to describe the prescribing indicators [12]. In a single pharmacy outlet 30 consecutive prescriptions were selected on one single day. If the required number is not obtained within 1 day data were collected on two consecutive days. The final number of prescriptions planned to be surveyed from the 80 pharmacy outlets were 2400. The WHO/INRUD core drug use indicators (prescribing and patient-care) defined below were evaluated [12].

#### A. Prescribing indicators

1. Average number of medicines per encounter - calculated by dividing the total number of different medicines prescribed by the number of prescriptions surveyed (WHO recommended value – 1.6-1.8)
2. Percentage of encounters with an antibiotic - calculated by dividing the number of encounters in which an antibiotic was prescribed by the total number of encounters surveyed, multiplied by 100 (WHO recommended value – 20-26.8%)
3. Percentage of encounters with an injection - calculated by dividing the number of encounters in which an injection was prescribed by the total number of encounters surveyed, multiplied by 100 (WHO recommended value – 13.4-24.1%)
4. Percentage of medicines prescribed by generic name - calculated by dividing the number of medicines prescribed by generic name by total number of medicines prescribed, multiplied by 100 (WHO recommended value – 100%)
5. Percentage of medicines prescribed from essential medicine list (EML) - calculated by dividing number of medicines prescribed which are in the EML [20] by the total number of medicines prescribed, multiplied by 100 (WHO recommended value – 100%)

#### B. Patient-care indicators

1. Percentage of medicines actually dispensed - calculated by dividing number of medicines dispensed by the total number of medicines prescribed, multiplied by 100 (WHO recommended value – 100%). A medicine was considered to be dispensed if the generic medication was dispensed by the pharmacy, including when an alternative brand was substituted irrespective of brand prescribed.
2. Percentage of medicines actually labeled - calculated by dividing number of medicines labeled by the total number of medicines dispensed, multiplied by 100 (WHO recommended value – 100%)

The above prescribing indicators were evaluated in comparison to WHO recommended optimal values as shown above [12]. Zhang and Zhi developed an index system for the comprehensive evaluation and comparison of healthcare system [21]. For the calculation of indices of non-poly-pharmacy, rational antibiotic use and safe injection use the WHO optimal value was divided by the observed value. To obtain the indices of generic name, medicines from EML, index of actually dispensed drugs and index of labeling of drugs the observed value was divided by the WHO optimal value. The optimal index for all indicators was set as 1, where values closer to 1 indicated rational use. The Index of Rational Drug Prescribing (IRDP) was calculated by adding the index values of all prescribing indicators. In a similar fashion, the Index of Rational Patient-Care Drug Use (IRPCDU) was calculated by adding the index values of all patient care indicators. These values were used for comparisons district wise and between different categories of pharmacy outlets.

### Data collection and analysis

Data were collected by trained medical graduates by perusal of prescriptions and interviewing of pharmacists. Data were recorded in a self-designed form formulated by modifying the WHO ordinary form for prescribing indicators [12]. Reliability of the data was ensured by following the WHO guidelines and methods [12]. In addition to the WHO prescribing indicators, most commonly prescribed medicines were also analysed. Statistical Package for Social Sciences (IBM SPSS Statistics for Windows, version 14.0, Armonk, NY: IBM Corp.) was used for analysis of data. Descriptive statistics such as frequencies, percentages, mean and standard deviation were calculated. Differences among the pharmacy outlets and the different districts were established using student t-test, ANOVA or chi-square tests as appropriate. The manuscript reporting adheres to STROBE guidelines (Supplementary Table S2). The statistical significance was determined by a *p* value < 0.05.

## Results

A total of 2328 prescriptions were included in the present analysis (97.0%), which comprised of 559 prescriptions from ‘Rajya Osusala’ pharmacies, 711 from SPC Franchisee pharmacy and 1058 from private pharmacies. Seventy-two prescriptions with incomplete details were excluded. The number of prescriptions from each district and their distribution across the different categories of pharmacies are depicted in Table 1. Overall, the ten most commonly prescribed medicines were atorvastatin (*n* = 280; 3.9%), losartan (*n* = 229; 3.2%), metformin (*n* = 219; 3.0%), paracetamol (*n* = 193; 2.7%), omeprazole (*n* = 186; 2.6%), aspirin (*n* = 179; 2.5%), domperidone (*n* = 149; 2.0%), clopidogrel (*n* = 133; 1.8%), cetirizine (*n* = 119; 1.6%) and gliclazide (*n* = 110; 1.5%). The commonest antibiotic, anti-diabetic and anti-hypertensive prescribed were co-amoxyclav (*n* = 109; 1.5%), metformin (*n* = 219; 3.0%) and losartan (*n* = 229; 3.2%) respectively. Table 2 shows the 10 most commonly prescribed medicines in the different categories of pharmacies, which showed only a slight variation between them. The 100 most commonly prescribed medications overall (Supplementary Table S3) are included as supplementary material.

**Table 1:** District wise distribution of pharmacies and number of prescriptions collected

District	Number of prescriptions			Total	% from total planned
	Privately owned	State owned (‘Rajya Osusala’ – SPC)	SPC Franchisee		
1. Ampara	30	30	NA	60	100
2. Anuradhapura	30	30	29	89	98.9
3. Badulla	30	30	29	89	98.9
4. Batticaloa	60	NA	NA	60	100
5. Colombo	55	60	57	172	95.6
6. Galle	60	60	58	178	98.9
7. Gampaha	60	59	57	176	97.8
8. Hambantota	29	25	28	82	91.1
9. Jaffna	30	30	25	85	94.4
10. Kalutara	59	58	56	173	96.1
11. Kandy	60	30	57	147	98.0
12. Kegalle	30	NA	28	58	96.7
13. Kilinochchi	58	NA	NA	58	96.7
14. Kurunegala	55	29	57	141	
15. Mannar	60	0	0	60	100
16. Mullaitivu					
17. Matale	30	NA	28	58	96.7
18. Matara	30	30	29	89	98.9
19. Monaragala	30	NA	28	58	96.7
20. Nuwara Eliya	57	NA	NA	57	95.0
21. Polonnaruwa	30	30	29	89	98.9
22. Puttalam	30	NA	29	59	98.3
23. Ratnapura	57	58	59	174	96.7
24. Trincomalee	30	NA	28	58	96.7
25. Vavuniya	58	NA	NA	58	96.7
Total	1058	559	711	2328	97.0

*NA* Not available, *SPC* State Pharmaceuticals Corporation

**Table 2:** Top 10 medications prescribed in the different categories of pharmacies

Medication	Overall	Number of times prescribed (Rank) % from total medications prescribed		
		Privately Owned	State owned (‘Rajya Osusala’ – SPC)	SPC Franchisee
Atorvastatin	280 (1) 3.9%	116 (1) 3.8%	106 (1) 5.3%	58 (4) 2.4%
Losartan	229 (2) 3.2%	82 (5) 2.7%	83 (2) 4.1%	64 (2) 2.9%
Metformin	219 (3) 3.0%	95 (3) 3.1%	67 (4) 3.3%	57 (5) 2.6%
Paracetamol	193 (4) 2.7%	99 (2) 3.3%	28 (9) 1.4%	66 (1) 3.0%
Omeprazole	186 (5) 2.6%	85 (4) 2.8%	39 (8) 1.9%	62 (3) 2.8%
Aspirin	179 (6) 2.5%	72 (6) 2.4%	75 (3) 3.7%	32 (10) 1.4%
Domperidone	149 (7) 2.0%	NA	43 (7) 2.1%	55 (6) 2.5%
Clopidogrel	133 (8) 1.8%	NA	55 (5) 2.7%	34 (9) 1.5%
Cetirizine	119 (9) 1.6%	54 (10) 1.8%	NA	44 (7) 2.0%
Gliclazide	110 (10) 1.5%	43 2.4%	47 (6) 2.3%	NA
Amoxycillin	NA	56 (8) 1.9%	NA	NA
Co-amoxyclav	NA	55 (9) 1.8%	NA	42 (8) 1.9%
Diclofenac sodium	NA	70 (7) 2.3%	NA	NA
Furosemide	NA	NA	25 (10) 1.2%	NA

NA - Medications not in the top 10 in given category, *SPC* State Pharmaceuticals Corporation

### Medicines per encounter

A total of 7255 medicines were prescribed in all the prescriptions, with the highest number being observed in prescriptions received at private pharmacies (3021), followed by SPC franchisee pharmacies (2217) and ‘Rajya Osusala’ pharmacies (2017). Overall, the average number of medicines per encounter was 3.1 ± 1.9 (Median: 3; range 1–12) (Table 3). The highest average number of medicines per encounter was reported in prescriptions received at ‘Rajya Osusala’ pharmacies (3.6 ± 2.2), a value which was significantly higher than other categories of pharmacies (*p* < 0.001). In the district wise analysis, the highest average number of medicines per encounter was reported from Kurunegala district (4.0 ± 2.0), followed by Rathnapura (3.9 ± 1.8) and Kalutara (3.8 ± 2.1), while the lowest average number of medicines per encounter was reported from Kilinochchi district (1.5 ± 0.6) (Supplementary Table S4). The average number of medicines per prescription was higher than the WHO optimal cut-off values (1.6–1.8), across all three categories of pharmacies and in all districts except the Kilinochchi district. The index for non-polypharmacy overall, in the private pharmacies, ‘Rajya Osusala’ pharmacies and SPC Franchisee pharmacies were 0.55, 0.59, 0.47 and 0.55 respectively (Table 4); with values ranging from 0.43 in Kurunegala district (lowest) to 1.00 (highest) in the Kilinochchi district (Supplementary Table S5).

**Table 3:** WHO/INRUD core drug use indicators overall and in the different categories of pharmacies

Prescribing Indicator (WHO recommended standard)	Mean ± SD (Median; Range) / Number (%)			
	Overall	Privately owned	State owned (‘Rajya Osusala’ – SPC)	SPC Franchisee
Prescribing Indicators				
1. Average medicines per encounter (1.6–1.8)	3.1 ± 1.9 (3; 1–12)	2.9 ± 1.7 (3; 1–11)^a^	3.6 ± 2.2 (3; 1–11)^a^	3.1 ± 1.9 (3; 1–12)^a^
2. Encounters with an antibiotic (%) (20–26.8%)	553 (23.8)	287 (27.1)^b^	84 (15.0)^ab^	182 (25.6)^a^
3. Encounters with an injection (%) (13.4–24.1%)	29 (1.2)	19 (1.8)^a^	6 (1.1)	4 (0.6)^a^
4. Medicines prescribed in generic name (%) (100%)	2579 (35.5)	980 (32.4)^b^	819 (40.6)^ba^	780 (35.2)^a^
5. Medicines prescribed from EML (%) (100%)	4991 (68.8)	2099 (69.5)	1368 (67.8)	1524 (68.7)
Patient-care Indicators				
1. Medicines actually dispensed (100%)	6701 (92.4)	2824 (93.5)^a^	1811 (89.8)^ab^	2066 (93.2)^b^
2. Medicines accurately labelled (100%)	6600 (98.5)	2758 (97.7)	1800 (99.4)	2042 (98.8)

^ab^ − values in a row with same symbols are significantly different from one another, *EML* Essential medicines list, *SD* Standard deviation, *SPC* State Pharmaceutical Corporation

**Table 4:** Index of Rational Drug Prescribing (IRDP) and Index of Rational Patient-Care Drug Use (IRPCDU) across the different categories of pharmacies

	Overall	Privately owned	State owned (‘Rajya Osusala’ – SPC)	SPC Franchisee
Prescribing Indicators				
1. Index of non-polypharmacy^a^	0.55	0.59	0.47	0.55
2. Index of rational antibiotic use^b^	0.98	0.86	1.00	0.91
3. Index of safe injection use^c^	1.00	1.00	1.00	1.00
4. Index of generic prescribing^d^	0.36	0.32	0.41	0.35
5. Index of EML prescribing^d^	0.69	0.70	0.68	0.69
Index of rational drug prescribing (1 + 2 + 3 + 4 + 5)	3.58	3.47	3.56	3.50
Patient-care Indicators				
1. Index of actually dispensed drugs^d^	0.92	0.94	0.90	0.93
2. Index of labeling of drugs^d^	0.98	0.98	0.99	0.99
Index of Rational Patient-Care Drug Use (1 + 2)	1.90	1.92	1.89	1.92

Optimal value taken as^a^1.7, ^b^23.4, ^c^18.75, ^d^100; EML – essential medicines list

### Prescribing antibiotics and encounters with an injection

The percentage of encounters with an antibiotic overall was 23.8% (*n* = 553). It was significantly lower in prescription received at 'Rajya Osusala’ pharmacies (15.0%), compared to the other two categories (*p* < 0.001) (Table 3). The highest percentage of encounters with an antibiotic was reported in prescriptions from the Mannar/Mullaitivu districts, which was 45.0%, followed by Trincomalee (39.7%) and Kilinochchi (36.2%) districts, while the lowest percentage was reported from the Kegalle district (8.6%) (Supplementary Table S4). The percentage of prescriptions with antibiotics did not exceed the WHO optimal cut-off values (20–26.8%) in most districts, except the districts of Badulla, Batticaloa, Nuwara-Eliya, Hambanthota, Kandy, Kilinochchi, Mannar, Monaragala, Puttalam, Trincomalee. However, the optimal cut-off value was exceeded only slightly in most of the above as well. The index for rational antibiotic use overall was 0.98; with the lowest value being observed in prescriptions from the Mannar/Mullaitivu districts (0.52) (Supplementary Table S5).

Overall, the percentage of encounters with an injection was only 1.2% (*n* = 29), with a significantly higher percentage being from prescriptions received at private pharmacies (1.8%), in comparison to SPC Franchisee pharmacies (*p* < 0.05) (Table 3). Encounters with an injection was only observed in 12 districts, with the highest being from Badulla district (5.6%), followed by Monaragala district (5.2%) (Supplementary Table S4). The percentage of prescriptions with injections was lower than the WHO optimal cut-off values (13.4–24.1%), overall, in the different categories of pharmacies and in all the districts evaluated. Index for safe injection use in overall and in the different categories of pharmacies are shown in Table 4, while the district wise analysis is shown in Supplementary Table S5.

### Generic name prescribing, essential medicines and IRDP

Overall, the percentage of medicines prescribed by generic name was only 35.5%, being highest in prescriptions that were received at the ‘Rajya Osusala’ pharmacies (40.6%), a value which was significantly higher than the other two categories of pharmacies (Table 3). The percentage of medicines prescribed by generic name ranged from 17.5% in Kegalle district to 62.4% in Kilinochchi district (Supplementary Table S4). Furthermore, except for the districts of Kilinochchi and Jaffna, percentage of medicines prescribed by generic name was < 50.0%. The percentage of medicines prescribed by generic name was lower than the WHO optimal cut-off values (100%) in all districts. Index of generic prescribing was 0.36 overall (Table 3), with the lowest being from Kegalle district (0.18) (Supplementary Table S5). The overall percentage of medicines prescribed from essential medicine list (EML) was 68.8%, without significant variation between the different categories of pharmacies (Table 3). In the district wise analysis, it was > 50.0% in all the districts evaluated, with the highest percentages identified from Mullaitivu and Mannar districts (91.2%) (Supplementary Table S4). However, the percentage of EML medicines was lower than the WHO optimal cut-off values (100%) in all the districts, with the overall index for EML prescribing being only 0.69 (Table 4), and the district wise values ranging from 0.54 (Ampara district) to 0.91 (Mullaitivu and Mannar districts) (Supplementary Table S5). The IRDP calculated by adding the index values of all prescribing indicators (Minimum 0; Maximum 5) was 3.58 overall and was highest in the ‘Rajya Osusala’ pharmacies (Table 4). In the district wise analysis, the IRDP was highest in the Kilinochchi district (4.11), being the only district with an IRDP value > 4.00, and lowest in Trincomalee district (3.14) (Supplementary Table S5).

### Patient care indicators and IRPCDU

Overall, the percentage of medicines actually dispensed was 92.4%, being highest in the private pharmacies (93.5%) (Table 3). In district-wise comparison, the Matale and Trincomalee districts (100%) had the highest percentage of medicines dispensed, while it was the lowest in the Monaragala district (76.3%) (Supplementary Table S4). The percentage of medicines actually dispensed was lower than the WHO optimal cut-off value (100%) in all except 2 districts. The index of actually dispensed drugs was 0.92 overall (Table 4) and ranged from 0.76 to 1.00 in the different districts (Supplementary Table S5). The percentage of medicines accurately labelled was 98.5%, being highest in the ‘Rajya Osusala’ pharmacies (99.4%) (Table 3). A high level of accurate labelling was observed with the percentage being > 95% in all districts. However, the WHO optimal cut-off value (100%) was achieved in only 10 districts (Supplementary Table S4). The data on index of labeling of drugs are presented in Table 4 and Supplementary Table S5. The Index of Rational Patient-Care Drug Use (IRPCDU) (Range 0 to 2) was 1.90 overall (Table 4), and was lowest in the Matara district (1.76), while being the highest (1.99) in the districts of Galle, Kurunegala and Mannar (Supplementary Table S5).

## Discussion

This is the first comprehensive evaluation of core drug use indicators in private sector pharmacies covering all 25 districts in Sri Lanka, with comparisons being made between the different categories of pharmacies and districts. In addition, the study surveyed more than 2,300 individual prescriptions, a number large enough to draw reasonable conclusions about WHO/INRUD core drug use indicators with the sample which is probably representing prescribing practices in primary health care. Furthermore, South Asia also known as the Indian sub-continent is home to about one-fourth of the world’s population, making it the most populous geographical regions in the world, and countries in the region share similar socio-economic standards and health care facilities. Hence, comprehensive evaluation of core drug use indicators, similar to the present study will be very important to appreciate existing practices and encourage the rational use of medicines in the Indian sub-continent. Table 5 provides a comparison of the summary findings from the present study with those of other developing countries from South Asia and other regions of the world. This provides a better understanding of the findings from the present evaluation in contrast to the regional and global context. The table includes countries from the African region (review) [22], Bahrain [23], Brazil [24], China [25], Jordan [26, 27], Pakistan [28] and Saudi Arabia [29, 30].

**Table 5:** WHO/INRUD core drug use indicators in different countries

Country [Ref]	Type of survey, Year	WHO/INRUD Core Drug use Indicators (WHO recommended value)						
		Prescribing Indictors					Patient Care Indicators	
		Medicines^*^ (1.6–1.8)	Antibiotics^†^ (20–26.8%)	Injections^‡^ (13.4–24.1%)	Generic name^¥^ (100%)	Essential Medicines^¶^ (100%)	Dispensed^#^ (100%)	Labelled^§^ (100%)
Sri Lanka	Present study, 2015	3.1	23.8%	1.2%	35.5%	68.8%	92.4%	98.5%
Africa	Region (PC), 2006–2015	3.5	49.0%	24.8%	70.4%	88.9%	NR	NR
Bahrain	PC, 2003	2.6	26.2%	8.3%	14.3%	99.8%	NR	NR
Brazil	National (PC), 2015	2.4	5.8%	6.0%	NR	45.1%	NR	NR
China	Provincial (PC), 2009–2010	3.2	50.9%	24.4%	NR	68.3%	NR	NR
Jordan	PC, 1999–2000	2.3	60.9%	1.2%	5.1%	93.0%	81.8%	91.4%
Pakistan	PC, 2014	3.4	48.9%	27.1%	71.6%	93.4%	90.9%	100%
Saudi Arabia	PC, 2010	2.4	32.2%	2.0%	61.2%	99.2%	99.6%	10.0%

^#^^*^Medicines per encounter; ^†^Encounters with an antibiotic; ^‡^Encounters with an injection; ^¥^Medicines prescribed in generic name; ^¶^Medicines prescribed from essential medicines list; ^#^Medicines actually dispensed; ^§^Medicines accurately labelled; *NR* Not reported; *PC* Primary Care

The average number of medicines per encounter was 3.1, which was higher than the WHO recommended value (1.6–1.8), being highest in prescriptions received at 'Rajya Osusala' pharmacies. The tendency of patients with chronic diseases who require a higher number of medicines have to go to state owned 'Rajya Osusala' outlets, expecting more affordable medicines with reasonable pricing may explain the above observation. Furthermore, the number of medicines per encounter was higher than the WHO optimal value in all districts in the country, except in Kilinochchi. However, as expected the average number of medicines per encounter was considerably lower compared to what has previously been observed in a Sri Lankan tertiary health care setting (4.8) [31]. In comparison to other countries the value was higher than those observed in middle eastern countries and Brazil, whilst being similar to China and lower than Pakistan (Table 5). A high number of medicines per prescription could indicate the presence of polypharmacy, which is generally considered when > = 4 medicines are prescribed in a prescription. In the present analysis, 35.3% of the prescriptions had more than 4 medicines per prescription. Increasing prevalence of non-communicable diseases in Sri Lanka with an aging population and the co-existent nature of these diseases are possibly contributing towards the higher number of medicines per prescription noted in the present study [32].. Adverse consequences of polypharmacy are well known. These include decreased adherence to therapy, increased adverse effects and medication errors, while imposing an unnecessary financial burden on both the patient and the healthcare system [33]. Therefore, urgent implementation of local evidence-based and rationalized policies to reduce polypharmacy is vital to minimize the harm caused by it.

The usage of antibiotics (23.8%) was within the WHO optimal values (20–26.8%) with most districts conforming to these standards. The antibiotic usage was found to be more than 30.0% in only 5 of the districts. Furthermore, the usage of antibiotics was lower than that of most of the other countries except Brazil (Table 5). This is an encouraging observation of reduced arbitrary use of antibiotics. However, selective interventions would help to further rationalize the usage of antibiotics within the country, especially in target districts such as Trincomalee, Kilinochchi, Mannar and Mullaitivu where > 35% of prescribing encounters contained antibiotics. The observation of significantly higher number of antibiotic prescriptions received in private pharmacies (27%) compared to 'Rajya Osusala' pharmacies (15%) may indicate the strict implementation of the need for a proper prescription to dispense antibiotics by the ‘Rajya osusala’ outlets (Table 3).

The usage of injectable preparations also showed a high degree of conformity with WHO standards, overall and in all districts studied, with only 10 districts having prescriptions that contain injections. The observed value of 1.2% in Sri Lanka is similar to Jordan, and the lowest among other countries compared (Table 5). This could be partly explained by the study sample representing mostly out-patient department prescriptions, while patients who require injections are generally admitted to health care institutions unless it is a regular, easily administered injection such as insulin. The excessive use of injections in countries like China (24.4%) and Pakistan (27.1%) is likely to have financial implications, whilst increasing the likelihood of iatrogenic infections and adverse effects [34]. Therefore, it is important to maintain the present conformity to accepted standards in the usage of injectable medicine and antibiotics in the local study setting, by conducting frequent and judicious reviews of prescribing practices.

In contrast, prescribing by generic name showed very poor compliance to the WHO recommended standards, with only 35.5% of the overall medicines being prescribed in their generic names. A limited Sri Lankan private sector survey conducted in 2002 also showed that only 36.7% of medicines are prescribed by generic name [35], a finding which also indicates that no significant change in practices have occurred during the last 10–15 years. Furthermore, in the district wise analysis the value was < 50.0% in all except two districts, being lowest in the Kegalle (17.5%) and Puttalam (17.8%) districts. According to available literature, generic prescribing is better in public health care settings, which has also been demonstrated in Sri Lanka where 90.1% medicines were prescribed by generic name in a public tertiary care hospital setting [22, 31]. Although the observed values were higher than in Bahrain and Jordan, they were considerably lower than most other countries in the South Asian region including Pakistan (71.6%) (Table 5). Generic name prescribing helps to improve communications among healthcare providers while minimizing the financial burden of the patient and reducing medication errors [36]. Therefore, national level policy decisions and strategies are necessary to strongly encourage generic prescribing in Sri Lankan. A recent such policy includes the recently implemented National Medicines Regulatory Authority (NMRA) act of Sri Lanka (2015) which makes it compulsory to write the generic name when prescribing [37]. The lack of confidence of the prescribers on the quality of generic medicines and not giving the liberty to the pharmacists to dispense any high-cost brand of the medicines are often stated as reasons for prescribers using brand names during prescribing. Therefore, NMRA act allowed to indicate a specific brand if desired after generic name and the pharmacist to inform the cost of all brands when dispensing medicines when prescriptions are written only using generic name. The impact of such initiatives needs to be evaluated through future surveys as this survey was conducted before the implementation of the NMRA act. The situation may have changed now. In contrast, prescribing medicines from the EML was better being 68.6% overall and > 50.0% in all of the districts. However, it did not conform to the WHO recommended standard (100%) and was significantly lower than most of the other countries except Brazil (Table 5). The concept of EML use is built on the principle that the use of a limited number of well-known and cost-effective medicines can lead to better health care, enhanced long-term medicines supply and more equitable and sustainable access to products [36]. Therefore, national level policies and implementation of initiatives are necessary to encourage Sri Lankan prescribers to use medicines from the EML.

When looking at the WHO/INRUD patient care indicators, the percentage of medicines actually dispensed was 92.4% (WHO recommended value 100%), with the highest being observed in private sector pharmacies. In all except 12 districts it was < 100%, with the lowest being reported from Monaragala district (76.3%). Although our value is higher than reported in Jordan and Pakistan, it was significantly lower than in Saudi Arabia (Table 5). Inadequate availability of drugs in stock is likely the main reason involved in the low percentage of actually dispensed drugs, together with increased non-generic prescribing, resulting in particular brands being unavailable when specifically requested by either the patient or the prescriber. Furthermore, the variations observed between districts could possibly point towards the non-equitable distribution of health resources within the country. The percentage of medicines accurately labelled was 98.5%, being highest in the government owned 'Rajya Osusala' pharmacies, whilst being > 95% in all the districts. However, the WHO recommended optimal value (100%) was only achieved in 10 districts. The WHO recommends that dispensed drugs should be accurately labeled with patient’s name, dose of the drug and regimen [28], which helps in the clear unambiguous identification of the medicine and its safe use. Therefore, shortcomings in labelling should be specifically addressed at places where it has been found to be deficient to further improve the quality of patient care.

Comparison of the different categories of pharmacies using the IRDP indicates that 'Rajya Osusala' pharmacies had the highest value (3.56), with only a slight difference between the different categories. In the district wise analysis, the IRDP ranged from 3.14 in Trincomalee district to 4.24 in Kilinochchi district, which was the only district with an acceptable level of rational medication use (IRDP> 4). Further improvements are necessary in all the other districts, especially in those which are below the national IRDP average of 3.58. In contract, the IRPCDU showed acceptable values, ranging from 1.49 to 1.79 in the different districts, although there is space for further improvement in specific districts. The IRDP and IRPCU, together with the district wise comparisons will benefit policy makers in prioritizing and implementing service improvement strategies. The differences observed between districts, especially in relation to Prescribing Indicators are possibly related to differences in population densities and resultant changes in health care facilities available. For example, districts such as Colombo, Kandy and Kurunegala, with higher population densities will also have more private healthcare institutions and hospitals, resulting in a higher number of patients with complex chronic diseases seeking treatment via these facilities, with a resultant increase in medications per encounter. Conversely comparatively resource poor districts could be affected by lesser penetration and implementation of evidence based practices and recommended guidelines, translating to higher encounters with antibiotics and reduced prescribing in generic name.

We also identified the list of most commonly prescribed medicines, which will be useful for prioritising medications during the teaching of medical and allied health sciences undergraduates. The strengths of the present survey include the comprehensive review of more than 2300 prescriptions from each of the 25 districts in Sri Lanka and across all the different categories of pharmacies present. Therefore, the findings are generalizable to the entire country. However, important limitations should also be acknowledged. We only evaluated two patient care indicators and did not assess other indicators such as the consultation time, dispensing time and patients’ knowledge, which would have helped to identify specific sources of the deficiencies noted. For example, dispensing time could be directly related to the accuracy of labelling. Furthermore, since seasonal variations in prescribing can have an impact on the prescribing indicators, the WHO recommends that data for prescribing should be collected over extended periods or in an inclusive manner covering different seasons [36]. However, Sri Lanka is a temperate country, with minimal seasonal variations, which are therefore unlikely any significant impact on prescribing patterns.

## Conclusions

This first comprehensive evaluation of core drug use indicators in private sector pharmacies in Sri Lanka which covered all 25 districts compared internationally comparable drug use indicators between the different categories of pharmacies in among all districts. We found that average number of medicines per encounter overall was higher than the WHO recommended value with polypharmacy detected in 35% prescriptions islandwide. This is possibly indicative of the high prevalence of non-communicable diseases in the country. It was heartening to note the usage of antibiotics and injectable drugs being within the WHO recommended values and comparing favourably with the values reported from other countries. Although the situation may have changed with enforcement of recent new legislations, one of the least acceptable indicators identified in this survey was in generic prescribing. The EML prescribing, labelling and percentage of dispensed medicines fared much better, though being lower than the WHO recommended 100% compliance. This island wide study has provided national wide data before the implementation of the important changes in regulation of medicines with the new NMRA ACT of 2015 and a repeat survey will be very useful to identify the impact of implementation of the new Act.

## Supplementary Information

**Additional file 1: Supplementary Table 1.** District wise distribution of pharmacies.

**Additional file 2: Supplementary Table S2.** STROBE Checklist for cross-sectional studies.

**Additional file 3: Supplementary Table S3.** Hundred most prescribed medicines in Sri Lankan pharmacies.

**Additional file 4: Supplementary Table S4.** WHO/INRUD core drug use indicators in the different districts.

**Additional file 5: Supplementary Table S5.** Index of Rational Drug Prescribing (IRDP) and Index of Rational Patient-Care Drug Use (IRPCDU) in the different districts.

## Data Availability

The datasets used and/or analysed during the current study available from the corresponding author on reasonable request.

## Abbreviations

EML: Essential Medicine List
GDP: Gross Domestic Product
HAI: Health Action International
INRUD: International Network of Rational Use of Drugs
IRDP: Index of Rational Drug Prescribing
IRPCDU: Index of Rational Patient-Care Drug Use
NMRA: National Medicines Regulatory Authority
SPC: State Pharmaceuticals Corporation
WHO: World Health Organization
