# Supplementary material for: WHO/INRUD Core drug use indicators and commonly prescribed medicines: a National Survey from Sri Lanka
Source: BMC Pharmacol Toxicol. 2021 Oct 28;22:67. doi: 10.1186/s40360-021-00535-5 (PMC8555184; doi:10.1186/s40360-021-00535-5)
Supplement: Supplementary file 3 — Additional file 3: Supplementary Table S3. Hundred most prescribed medicines in Sri Lankan pharmacies. [file 40360_2021_535_MOESM3_ESM.docx]

**Supplementary Table S3**: Hundred most prescribed medicines in Sri Lankan pharmacies (N=7,255)

| Medication | Number of  times prescribed | Percentage |
| --- | --- | --- |
| 1. Atorvastatin | 280 | 3.9 |
| 1. Losartan | 229 | 3.2 |
| 1. Metformin | 219 | 3.0 |
| 1. Paracetamol | 193 | 2.7 |
| 1. Omeprazole | 186 | 2.6 |
| 1. Aspirin | 179 | 2.5 |
| 1. Domperidone | 149 | 2.0 |
| 1. Clopidogrel | 133 | 1.8 |
| 1. Cetirizine | 119 | 1.6 |
| 1. Gliclazide | 110 | 1.5 |
| 1. Diclofenac sodium | 109 | 1.5 |
| Co-amoxyclav | 109 | 1.5 |
| 1. Pantoprazole | 106 | 1.5 |
| 1. Amoxicillin | 101 | 1.4 |
| 1. Esomeprazole | 83 | 1.1 |
| 1. Chlorpheniramine | 80 | 1.1 |
| 1. Loratadine | 74 | 1.0 |
| 1. Metronidazole | 68 | 0.9 |
| 1. Celecoxib | 67 | 0.9 |
| 1. Ciprofloxacin | 66 | 0.9 |
| Rosuvastatin | 66 | 0.9 |
| 1. Diltiazem | 63 | 0.9 |
| 1. Furosemide | 61 | 0.8 |
| Paracetamol and Codeine | 61 | 0.8 |
| 1. Hydrochlorothiazide | 57 | 0.8 |
| Metoprolol | 57 | 0.8 |
| Multivitamin | 57 | 0.8 |
| 1. Prednisolone | 56 | 0.8 |
| 1. Azithromycin | 53 | 0.7 |
| 1. Diclofenac gel | 51 | 0.7 |
| 1. Famotidine | 50 | 0.7 |
| 1. Cefuroxime | 49 | 0.7 |
| 1. Folic acid | 46 | 0.6 |
| Vitamins and minerals | 46 | 0.6 |
| 1. Rabeprazole | 45 | 0.6 |
| 1. Enalapril | 43 | 0.6 |
| 1. Montelukast | 42 | 0.6 |
| 1. Fexofenadine | 41 | 0.6 |
| Theophylline | 41 | 0.6 |
| 1. Clarithromycin | 38 | 0.5 |
| 1. Betamethasone | 37 | 0.5 |
| 1. Cloxacillin | 35 | 0.5 |
| Etoricoxib | 35 | 0.5 |
| 1. Aceclofenac | 34 | 0.5 |
| Cefalexin | 34 | 0.5 |
| 1. Ibuprofen | 33 | 0.5 |
| Mefenamic acid | 33 | 0.5 |
| 1. Isosorbide mononitrate | 31 | 0.4 |
| 1. Nifedipine | 30 | 0.4 |
| 1. Amlodipine | 29 | 0.4 |
| Flunarizine | 29 | 0.4 |
| Pregabalin | 29 | 0.4 |
| 1. Glimepiride | 28 | 0.4 |
| 1. Beclomethasone | 27 | 0.4 |
| 1. Dexamethasone | 26 | 0.4 |
| Formoterol and Budesonide | 26 | 0.4 |
| Gabapentin | 26 | 0.4 |
| Methylprednisolone | 26 | 0.4 |
| 1. Glucosamine | 25 | 0.3 |
| 1. Ketotifen | 24 | 0.3 |
| 1. Meloxicam | 23 | 0.3 |
| Vitamin C | 23 | 0.3 |
| 1. Aluminum hydroxide and   magnesium trisilicate suspension | 21 | 0.3 |
| Thyroxine | 21 | 0.3 |
| Vitamin B complex | 21 | 0.3 |
| 1. Amitriptyline | 20 | 0.3 |
| Atenolol | 20 | 0.3 |
| Bisoprolol | 20 | 0.3 |
| Cefixime | 20 | 0.3 |
| Glyceryl trinitrate | 20 | 0.3 |
| 1. Aluminum magnesium   simethicone suspension | 19 | 0.3 |
| Captopril | 19 | 0.3 |
| Doxycycline | 19 | 0.3 |
| Hydrocortisone | 19 | 0.3 |
| Terbutaline | 19 | 0.3 |
| 1. Ferrous sulphate and folic acid | 18 | 0.2 |
| Pioglitazone | 18 | 0.2 |
| Risperidone | 18 | 0.2 |
| Vitamin D and calcium | 18 | 0.2 |
| 1. Benzhexol | 17 | 0.2 |
| Prazosin | 17 | 0.2 |
| 1. Betahistine | 16 | 0.2 |
| Ketotifen | 16 | 0.2 |
| Metoclopramide | 16 | 0.2 |
| Nicorandil | 16 | 0.2 |
| 1. Alfacalcidol | 15 | 0.2 |
| Salbutamol, bromhexine and guaifenesin | 15 | 0.2 |
| 1. Alprazolam | 14 | 0.2 |
| Levofloxacin | 14 | 0.2 |
| Mebendazole | 14 | 0.2 |
| Oral rehydration solution | 14 | 0.2 |
| Sitagliptin | 14 | 0.2 |
| Tamsulosin | 14 | 0.2 |
| Tramadol | 14 | 0.2 |
| 1. Carbamazepine | 13 | 0.2 |
| Clonazepam | 13 | 0.2 |
| Clotrimazole | 13 | 0.2 |
| Desloratadine | 13 | 0.2 |
| Emollient | 13 | 0.2 |
| Fenofibrate | 13 | 0.2 |
| Fusidic acid | 13 | 0.2 |
| Methyldopa | 13 | 0.2 |
