# Supplementary material for: WHO/INRUD Core drug use indicators and commonly prescribed medicines: a National Survey from Sri Lanka
Source: BMC Pharmacol Toxicol. 2021 Oct 28;22:67. doi: 10.1186/s40360-021-00535-5 (PMC8555184; doi:10.1186/s40360-021-00535-5)
Supplement: Supplementary file 4 — Additional file 4: Supplementary Table S4. WHO/INRUD core drug use indicators in the different districts. [file 40360_2021_535_MOESM4_ESM.docx]

**Supplementary Table S4:** WHO/INRUD core drug use indicators in the different districts

| Prescribing Indicator  (WHO recommended standard) | Mean ± SD / Number  (Median; Range) / (%) | | | | | | | | | | | | | | | | | | | | | | | |
| --- | --- | --- | --- | --- | --- | --- | --- | --- | --- | --- | --- | --- | --- | --- | --- | --- | --- | --- | --- | --- | --- | --- | --- | --- |
|  | Ampara | Anuradhapura | Badulla | Batticaloa | Colombo | Galle | Gampaha | Hambanthota | Jaffna | Kalutara | Kandy | Kegalle | Kilinochchi | Kurunegala | Mannar  Mullaitivu | Matale | Matara | Monaragala | Nuwara-Eliya | Polonnaruwa | Puttalam | Rathnapura | Trincomalee | Vavuniya |
| Prescribing Indicators   1. Average medicines per encounter (1.6-1.8) 2. Encounters with an antibiotic (%) (20-26.8%) 3. Encounters with an injection (%) (13.4-24.1%) 4. Medicines prescribed in generic name (%) (100%) 5. Medicines prescribed from EML (%) (100%) | 2.7±1.6  (2; 1-6)  16  (26.7)  1  (1.7)  45  (28.1)  86  (53.8) | 3.5±1.7  (4; 1-10)  19  (21.3)  0  130  (41.7)  183  (58.7) | 2.8±1.1  (3; 1-5)  26  (29.2)  5  (5.6)  59  (23.7)  188  (75.5) | 2.8±1.3  (3; 1-8)  17  (28.3)  0  65  (38.2)  92  (54.1) | 2.9±2.3  (2; 1-12)  39  (22.7)  4  (2.3)  219  (43.9)  339  (67.9) | 2.5±1.4  (2; 1-10)  32  (18.0)  3  (1.7)  165  (37.0)  326  (73.1) | 2.7±1.7  (2; 1-8)  41  (23.3)  1  (0.6)  199  (41.9)  329  (69.3) | 3.5±2.0  (3.5; 1-10)  24  (29.3)  1  (1.2)  125  (43.9)  221  (77.5) | 3.5±2.2  (3; 1-10)  16  (18.8)  0  152  (51.5)  210  (71.2) | 3.8±2.1  (3; 1-11)  38  (22.0)  5  (2.9)  229  (35.2)  414  (63.6) | 3.8±2.3  (3; 1-11)  48  (32.7)  0  221  (39.4)  415  (74.0) | 2.4±1.3  (2; 1-6)  5  (8.6)  0  24  (17.5)  96  (70.1) | 1.5±0.6  (1; 1-3)  21  (36.2)  0  53  (62.4)  71  (83.5) | 4.0±2.0  (4; 1-10)  24  (17.0)  1  (0.7)  132  (23.5)  346  (61.7) | 3.2±1.2  (2; 1-6)  27  (45.0)  2  (3.3)  68  (35.1)  177  (91.2) | 2.2±1.2  (2; 1-5)  15  (25.9)  0  42  (33.1)  91  (71.7) | 3.5±1.9  (3; 1-10)  12  (13.5)  0  150  (48.2)  221  (71.1) | 2.4±1.6  (3; 1-8)  17  (29.3)  3  (5.2)  50  (36.0)  93  (66.9) | 1.9±1.0  (2; 1-4)  16  (28.1)  0  39  (35.5)  74  (67.3) | 3.1±1.7  (3; 1-10)  20  (22.5)  0  101  (36.3)  199  (71.6) | 3.4±1.8  (3; 1-10)  18  (30.5)  0  36  (17.8)  142  (70.3) | 3.9±1.8  (4; 1-10)  25  (14.4)  0  183  (27.0)  448  (66.2) | 3.3±1.5  (4; 1-7)  23  (39.7)  2  (3.4)  39  (20.4)  158  (82.7) | 2.4±1.8  (2; 1-8)  14  (24.1)  1  (1.7)  53  (37.9)  103  (73.6) |
| Patient-care Indicators   1. Medicines actually dispensed (100%) 2. Medicines accurately labelled (100%) | 137  (85.6)  136  (99.3) | 294  (94.2)  283  (96.3) | 244  (98.0)  238  (97.5) | 153  (90.6)  148  (96.1) | 435  (87.2)  430  (98.9) | 440  (98.7)  440  (100) | 432  (90.9)  415  (96.1) | 258  (90.5)  252  (97.7) | 286  (96.9)  286  (100) | 594  (91.2)  570  (96.0) | 518  (92.3)  518  (100) | 132  (96.4)  132  (100) | 78  (91.8)  78  (100) | 556  (99.1)  554  (99.6) | 187  (96.4)  187  (100) | 127  (100)  126  (99.2) | 288  (92.6)  288  (100) | 106  (76.3)  106  (100) | 104  (94.5)  104  (100) | 266  (95.7)  266  (100) | 177  (87.6)  175  (98.9) | 576  (85.1)  569  (98.8) | 191  (100)  182  (95.3) | 121  (86.4)  120  (99.2) |
