# Supplementary material for: WHO/INRUD Core drug use indicators and commonly prescribed medicines: a National Survey from Sri Lanka
Source: BMC Pharmacol Toxicol. 2021 Oct 28;22:67. doi: 10.1186/s40360-021-00535-5 (PMC8555184; doi:10.1186/s40360-021-00535-5)
Supplement: Supplementary file 5 — Additional file 5: Supplementary Table S5. Index of Rational Drug Prescribing (IRDP) and Index of Rational Patient-Care Drug Use (IRPCDU) in the different districts. [file 40360_2021_535_MOESM5_ESM.docx]

**Supplementary Table S5:** Index of Rational Drug Prescribing (IRDP) and Index of Rational Patient-Care Drug Use (IRPCDU) in the different districts

|  | Ampara | Anuradhapura | Badulla | Batticaloa | Colombo | Galle | Gampaha | Hambanthota | Jaffna | Kalutara | Kandy | Kegalle | Kilinochchi | Kurunegala | Mannar  Mullaitivu | Matale | Matara | Monaragala | Nuwara-Eliya | Polonnaruwa | Puttalam | Rathnapura | Trincomalee | Vavuniya |
| --- | --- | --- | --- | --- | --- | --- | --- | --- | --- | --- | --- | --- | --- | --- | --- | --- | --- | --- | --- | --- | --- | --- | --- | --- |
| Prescribing Indicators   1. Index of non-polypharmacy^*^ | 0.63 | 0.49 | 0.61 | 0.61 | 0.59 | 0.68 | 0.63 | 0.49 | 0.49 | 0.45 | 0.45 | 0.71 | 1.00 | 0.43 | 0.53 | 0.77 | 0.49 | 0.71 | 0.89 | 0.55 | 0.50 | 0.44 | 0.52 | 0.71 |
| 1. Index of rational antibiotic use^†^ | 0.88 | 1.00 | 0.80 | 0.83 | 1.00 | 1.00 | 1.00 | 0.80 | 1.00 | 1.00 | 0.72 | 1.00 | 0.65 | 1.00 | 0.52 | 0.90 | 1.00 | 0.80 | 0.83 | 1.00 | 0.77 | 1.00 | 0.59 | 0.97 |
| 1. Index of safe injection use^‡^ | 1.00 | 1.00 | 1.00 | 1.00 | 1.00 | 1.00 | 1.00 | 1.00 | 1.00 | 1.00 | 1.00 | 1.00 | 1.00 | 1.00 | 1.00 | 1.00 | 1.00 | 1.00 | 1.00 | 1.00 | 1.00 | 1.00 | 1.00 | 1.00 |
| 1. Index of generic prescribing^#^ | 0.28 | 0.42 | 0.24 | 0.38 | 0.44 | 0.37 | 0.42 | 0.44 | 0.52 | 0.35 | 0.39 | 0.18 | 0.62 | 0.24 | 0.35 | 0.33 | 0.48 | 0.36 | 0.35 | 0.36 | 0.18 | 0.27 | 0.20 | 0.38 |
| 1. Index of EML prescribing^#^ | 0.54 | 0.59 | 0.76 | 0.54 | 0.68 | 0.73 | 0.69 | 0.78 | 0.71 | 0.64 | 0.74 | 0.70 | 0.84 | 0.62 | 0.91 | 0.72 | 0.71 | 0.67 | 0.67 | 0.72 | 0.70 | 0.66 | 0.83 | 0.74 |
| Index of rational drug prescribing (1+2+3+4+5) | 3.33 | 3.50 | 3.41 | 3.36 | 3.71 | 3.78 | 3.74 | 3.51 | 3.72 | 3.44 | 3.30 | 3.59 | 4.11 | 3.29 | 3.31 | 3.72 | 3.68 | 3.54 | 3.74 | 3.63 | 3.15 | 3.37 | 3.14 | 3.80 |
| Patient-care Indicators |  |  |  |  |  |  |  |  |  |  |  |  |  |  |  |  |  |  |  |  |  |  |  |  |
| 1. Index of actually dispensed drugs^#^ | 0.86 | 0.94 | 0.98 | 0.91 | 0.87 | 0.99 | 0.91 | 0.91 | 0.97 | 0.91 | 0.92 | 0.96 | 0.92 | 0.99 | 1.00 | 0.93 | 0.76 | 0.96 | 0.95 | 0.96 | 0.88 | 0.85 | 1.00 | 0.86 |
| 1. Index of labeling of drugs^#^ | 0.99 | 0.96 | 0.98 | 0.96 | 0.99 | 1.00 | 0.96 | 0.98 | 1.00 | 0.96 | 1.00 | 1.00 | 1.00 | 1.00 | 0.99 | 1.00 | 1.00 | 1.00 | 1.00 | 1.00 | 0.99 | 0.99 | 0.95 | 0.99 |
| Index of Rational Patient-Care Drug Use (IRPCDU) | 1.85 | 1.90 | 1.96 | 1.87 | 1.86 | 1.99 | 1.87 | 1.89 | 1.97 | 1.87 | 1.92 | 1.96 | 1.92 | 1.99 | 1.99 | 1.93 | 1.76 | 1.96 | 1.95 | 1.96 | 1.87 | 1.84 | 1.95 | 1.85 |

Optimal value taken as *1.7, ^†^23.4, ^‡^18.75, ^#^100; EML – essential medicines list
